# Inhibition of Stress-Induced Viral Promoters by a Bovine Herpesvirus 1 Non-Coding RNA and the Cellular Transcription Factor, β-Catenin

**DOI:** 10.3390/ijms22020519

**Published:** 2021-01-07

**Authors:** Jing Zhao, Nishani Wijesekera, Clinton Jones

**Affiliations:** Department of Veterinary Pathobiology, College of Veterinary Medicine, Oklahoma State University, Stillwater, OK 74078, USA; zhaojingnh2005@163.com (J.Z.); nwijese@ostatemail.okstate.edu (N.W.)

**Keywords:** BoHV-1, stress, glucocorticoid receptor, maintenance of latency, latency-related gene, Wnt/β-catenin signaling pathway

## Abstract

The ability to establish, maintain, and reactivate from latency in sensory neurons within trigeminal ganglia (TG) is crucial for bovine herpesvirus 1 (BoHV-1) transmission. In contrast to lytic infection, the only viral gene abundantly expressed during latency is the latency-related (LR) gene. The synthetic corticosteroid dexamethasone consistently induces reactivation from latency, in part because the glucocorticoid receptor (GR) transactivates viral promoters that drive expression of key viral transcriptional regulator proteins (bICP0 and bICP4). Within hours after dexamethasone treatment of latently infected calves, LR gene products and β-catenin are not readily detected in TG neurons. Hence, we hypothesized that LR gene products and/or β-catenin restrict GR-mediated transcriptional activation. A plasmid expressing LR RNA sequences that span open reading frame 2 (ORF2-Stop) inhibited GR-mediated transactivation of the BoHV-1 immediate early transcription unit 1 (IEtu1) and mouse mammary tumor virus (MMTV) promoter activity in mouse neuroblastoma cells (Neuro-2A). ORF2-Stop also reduced productive infection and GR steady-state protein levels in transfected Neuro-2A cells. Additional studies revealed that the constitutively active β-catenin mutant reduced the transactivation of the IEtu1 promoter by GR and dexamethasone. Collectively, these studies suggest ORF2 RNA sequences and Wnt/β-catenin signaling pathway actively promote maintenance of latency, in part, by impairing GR-mediated gene expression.

## 1. Introduction

Bovine herpesvirus 1 (BoHV-1) infection frequently causes upper respiratory tract disease and erodes mucosal surfaces of the upper respiratory tract [1]. Hence, the virus-induced pathology in the respiratory tract promotes colonization of the Gram-negative bacterium, Mannheimia hemolytica (MH), in the lower respiratory tract and enhances interactions between the *MH* leukotoxin and bovine peripheral blood mononuclear cells [2,3]. Co-infection of calves with BoHV-1 and *MH* consistently leads to life-threatening pneumonia, which is a hallmark of bovine respiratory disease [4]. The discovery that the BoHV-1 receptor protein is a significant bovine respiratory disease susceptibility gene [5] supports the concept that BoHV-1 is a cofactor in this polymicrobial disease.

BoHV-1 establishes life-long latency in neurons [6,7]. If infection is initiated via the ocular, oral, or nasal cavity, sensory neurons in trigeminal ganglia (TG) are a primary site for latency. Following acute infection, latency is established, and the shedding of infectious virus is not detected by virus isolation approaches. However, viral genomes are present in neurons within the peripheral nervous system: hence, latency is established. The establishment and maintenance of latency can only occur if lytic cycle viral gene expression is restricted and neurons survive infection, as reviewed in [6,7]. The only viral gene abundantly expressed during the maintenance of latency is the latency-related (LR) RNA, which encodes at least two micro-RNAs and a protein open reading frame 2 (ORF2) [8]. ORF2 interferes with apoptosis and interacts with cellular proteins, including Notch 1, Notch 3, β-catenin, and CCAAT/enhancer-binding protein alpha [9,10,11]. Over-expression of these micro-RNAs reduces bICP0 protein expression [12], which is a crucial viral transcriptional regulatory protein. Thus, LR gene products are predicted to support the establishment and maintenance of latency in neurons.

The Wnt/β-catenin signaling pathway is differentially regulated during the BoHV-1 latency-reactivation cycle [11,13,14]. Two Wnt-associated cellular transcription factors—β-catenin and a β-catenin coactivator, high mobility group AT-hook 1 protein (HMGA1)—are readily detected in the TG neurons of latently infected but not uninfected calves or latently infected calves treated with the synthetic corticosteroid dexamethasone (DEX) to initiate reactivation from latency [13,14]. Interestingly, ORF2 interacts with a complex containing β-catenin and HMGA1 in transfected cells and enhances β-catenin dependent transcription and survival of neuroblastoma cells [14]. Canonical Wnt/β-catenin signaling promotes cell survival, axonal growth, and establishes proper axonal connections [15,16]. The increased expression of at least seven Wnt antagonists occurs during early stages of DEX-induced reactivation [11,13], which generally reduces neuronal survival [17].

Stress increases the incidence of BoHV-1 [18], herpes simplex virus 1 [18,19], and canine herpesvirus 1 reactivation from latency [20]. Within three hours after DEX treatment of latently infected calves, expression of specific cellular transcription factors are induced in TG neurons that stimulate productive infection and viral promoters [21]. Strikingly, the BoHV-1 genome contains more than 100 putative glucocorticoid receptor (GR) response elements (GREs) [22]. Two functional GREs are located in the immediate early transcription unit 1 (IEtu1) promoter [22,23]. This viral promoter drives the expression of two viral transcriptional regulators (bICP0 and bICP4) that are essential for productive infection [24,25].

Studies in this manuscript tested whether ORF2 and β-catenin interfere with stress-induced gene expression. An ORF2 expression construct that lacks the first in-frame methionine codon and contains stop codons at the amino-terminus of ORF2 impaired GR-mediated transactivation of the IEtu1 promoter and mouse mammary tumor virus (MMTV) long terminal repeat (LTR), in part by reducing GR steady-state protein levels. A β-catenin mutant protein (S33Y) resistant to proteasome-mediated degradation reduced the GR-mediated transactivation of IEtu1 promoter activity. Wild-type (wt) β-catenin, in part due to its rapid degradation, did not significantly stimulate the IEtu1 promoter. These studies suggest ORF2 RNA sequences support the maintenance of a latent infection. 

## 2. Results

### 2.1. ORF2 Sequences Reduce GR+DEX-Mediated Transactivation 

Mammals, including cattle, generally encounter low levels of stress every day; however, calves latently infected with BoHV-1 do not shed virus daily due to reactivation from latency. Support for this premise comes from the finding that DNAse I resistant BoHV-1 DNA is infrequently detected by PCR in ocular or nasal swabs from 2 weeks to 60 days after calves are experimentally infected (C Jones: unpublished studies). This observation suggests that viral and/or cellular factors prevent low levels of stress from stimulating viral gene expression and reactivation from latency. ORF2, a BoHV-1-encoded protein, is expressed in a subset of latently infected neurons [26] and is proposed to play a crucial role in the latency–reactivation cycle [27]. As a result of alternative splicing, ORF2 isoforms exist that have common sequences within the first ½ of the amino terminus; however, C-terminal sequences are variable [28].

**Figure 1:**
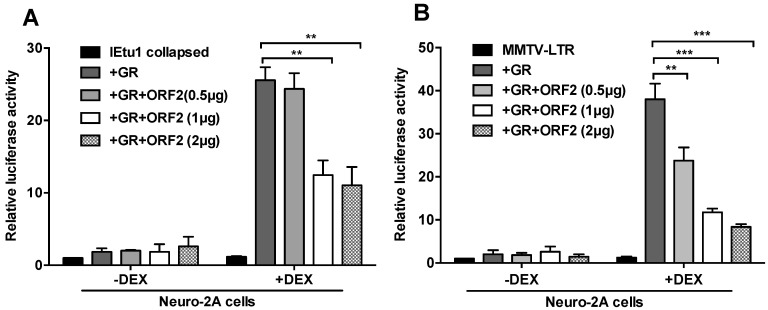
Open reading frame 2 (ORF2) inhibits glucocorticoid receptor (GR) and dexamethasone (DEX)-mediated transactivation of stress-inducible promoters. **Panel** (**A**): Neuro-2A cells were transfected with the IEtu1 collapsed promoter construct containing the firefly luciferase reporter gene (0.5 μg), GR expression plasmid (1.0 μg), and ORF2 expression plasmid (0.5, 1.0 or 2.0 μg). **Panel** (**B**): Neuro-2A cells were transfected with the mouse mammary tumor virus long terminal repeat (MMTV-LTR) promoter construct (0.5 μg) and wt ORF2 expression construct (0.5, 1.0, or 2.0 μg). All transfections contained a plasmid that expresses Renilla luciferase (0.05 μg). To maintain the same amount of DNA in each sample, an empty vector was included in certain samples. Cells were incubated with 2% stripped fetal bovine serum (FBS) 24 h after transfection and as denoted certain cultures treated with DEX (10 μM). At 48 h after transfection, cells were harvested, and protein lysate subjected to a dual-luciferase assay. The results are the average of three independent experiments and error bars denote the standard error. The student’s T test was used for statistical analysis: ns, not significant; **, *p* < 0.01; ***, *p* < 0.001.

Based on these observations, we hypothesized that ORF2 impairs stress-induced transcription. Genomic ORF2 sequences consisting of the ORF2 variant abundantly detected as a poly A+ RNA in latently infected calves were used for these studies [28]. Neuro-2A cells were used because they have neuronal-like properties and can be differentiated into dopamine-like neurons [29]. Furthermore, the infection of Neuro-2A cells with BoHV-1 yields low levels of infectious BoHV-1 [30], these cells are readily transfected, and ORF2 is consistently expressed in Neuro-2A cells [10,31,32]; whereas other common cell lines do not support the consistent expression of ORF2. The BoHV-1 IEtu1 collapsed promoter was used for these studies because this promoter contains two GR response elements (GRE) required for GR-mediated transactivation, and this construct is strongly transactivated by GR and DEX [22,23,33]. The ORF2 expression plasmid consistently reduced GR-mediated transactivation of the IEtu1 promoter in a dose-dependent manner when DEX was added to cultures after transfection (Figure 1A). Conversely, basal IEtu1 promoter activity (no DEX added to cultures) was not dramatically influenced by ORF2. 

The effects of the ORF2 construct on the mouse mammary tumor virus (MMTV) long terminal repeat (LTR) was also examined because this promoter is strongly stimulated by GR+DEX due to multiple GREs in the LTR [34,35]. The ORF2 expression plasmid significantly reduced GR-mediated transactivation of the MMTV LTR in Neuro-2A cells (Figure 1B). With respect to both IEtu1 collapsed and MMTV LTR promoters, the ORF2 expression construct reduced GR+DEX mediated transcription in a dose-dependent effect. 

### 2.2. ORF2 RNA Sequences Interfere with GR-Mediated Transactivation 

**Figure 2:**
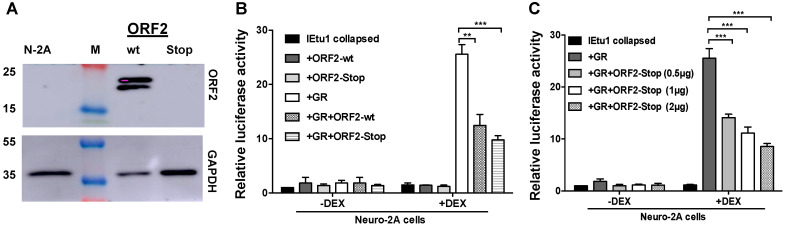
The construct of an ORF2 mutant that lacks the first in-frame methionine and contains stop codons at its amino-terminus (ORF2-Stop) reduced GR+DEX mediated transactivation of the immediate early transcription unit 1 (IEtu1) collapsed promoter. **Panel** (**A**): Neuro-2A cells were transfected with plasmids that express wt ORF2 (2.0 μg) or ORF2-Stop construct (2.0 μg). Forty-eight hours after transfection, whole cell lysate (WCL) was prepared and processed for Western blot analysis using a Flag-specific antibody to detect ORF2 or a GAPDH-specific antibody. Each lane contained 10 μg protein from WCL. GAPDH was measure as a loading control. Position of molecular weight markers are shown. **Panel** (**B**): Neuro-2A cells were transfected with the IEtu1 collapsed promoter construct (0.5 μg), GR (1.0 μg), wt ORF2 (1.0 μg), or ORF2-Stop construct (1.0 μg). **Panel** (**C**): Neuro-2A cells were transfected with the IEtu1 collapsed promoter construct (0.5 μg), GR (1.0 μg), and ORF2-Stop construct (0.5, 1.0, or 2.0 μg). Panels B and C: all transfections contained a plasmid that expresses Renilla luciferase (0.05 μg). To maintain the same amount of DNA in each sample, an empty vector was included in certain samples. Cells were incubated with 2% stripped FBS 24 h after transfection, and certain cultures were treated with DEX (10 μM). At 48 h after transfection, cells were harvested, and protein lysate was subjected to a dual-luciferase assay. The results are the average of three independent experiments, and error bars denote the standard error. The Student’s T test was used for statistical analysis: **, *p* < 0.01; ***, *p* < 0.001.

The ORF2 protein localizes to the nuclear rim, interferes with apoptosis [36], and preferentially interacts with single-stranded DNA but not RNA [32]. ORF2 also interacts with several cellular transcription factors, including Notch 1 and 3 [10], β-catenin [13], HMGA1 [14], and CCAAT enhancer binding protein alpha [37]. Prior to identifying ORF2 protein domains that impair GR-mediated transactivation, we examined an ORF2 mutant that lacks the first in-frame methionine and contains stop codons at its amino-terminus (ORF2-Stop). As expected, the wt ORF2 plasmid expressed a Flag-tagged protein that migrated at approximately 20 kd in transfected Neuro-2A cells (Figure 2A). The ORF2-Stop mutant does not express detectable levels of the ORF2 protein, which is consistent with previous studies [14,36]. However, ORF-2-Stop and wt ORF-2 constructs express ORF2 mRNA [36]. Two ORF-2 specific bands are consistently observed in Western blots, which may be due to the phosphorylation of ORF2, because this open reading frame contains several consensus protein kinase phosphorylation sites [8]. Strikingly, the ORF2-Stop mutant inhibited IEtu1 promoter activity in transfected Neuro-2A cells with similar efficiency as the wt ORF2 constructs (Figure 2B), suggesting a non-coding RNA spanning ORF2 coding sequences was important. Additional studies revealed RNA sequences that span ORF2 interfered with IEtu1 promoter activity in a dose-dependent manner (Figure 2C).

### 2.3. ORF2 Impairs Productive Infection

**Figure 3:**
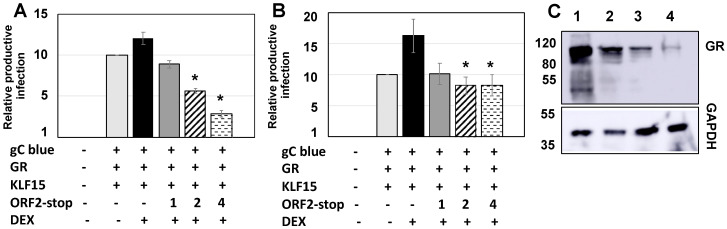
Latency-related (LR) ORF2 RNA sequences interfere with productive infection and reduce GR steady-state protein levels. **Panel** (**A**): Neuro-2A cells were transfected with 1.5 ug BHV-1 gC-Blue, and where indicated, a plasmid-expressing mouse GR protein (1.0 ug DNA), Krüppel-like transcription factor 15 (KLF15) (0.5 ug DNA), and ORF2-Stop (1, 2, or 4 ug plasmid). To maintain the same amount of DNA in each sample, an empty vector was included in samples. After transfection, 2% stripped FBS was added to media. Then, designated cultures were treated with water-soluble DEX (10 uM; Sigma; St. Louis, MO, USA). At 48 h after transfection, the number of β-Gal+ Neuro-2A cells was counted from four independent quadrants/plate. **Panel** (**B**): Rabbit skin (RS) cells were transfected with the designated plasmids as described in Panel A. At 24 h after transfection, the number of β-Gal+ RS cells was counted from four independent quadrants/plate. Twenty-four hours was used for RS cells because at later times after transfection, virus shedding occurs in the initial transfected cell and spread to surrounding cells, which culminates in disruption of the monolayer due to productive infection. For Panels A and B, the results are the average of three independent experiments. The value for the control (gC-Blue virus cotransfected with empty vector and then treated with PBS after transfection) was set at 1. An asterisk indicates a significant difference between control and samples transfected with the GR and/or KLF15 and treated with DEX (*p* < 0.05) using the Student *t*-test. **Panel** (**C**): Neuro-2A cells were cotransfected with the GR expression construct (1.0 ug DNA; lane 1) and 1 (lane 2), 2 (lane 3), or 4 (lane 4) ug of the ORF2-Stop plasmid (ug DNA). To maintain the same amount of DNA in each sample, an empty vector was included in certain samples. Cells were incubated with 2% stripped FBS 24 h after transfection, and cultures were treated with DEX (10 μM) until 48 h after transfection. Then, WCL was prepared, and Western blots were performed to measure GR: 10 ug WCL was used for each lane. Position of molecular weight markers are shown.

To test whether the over-expression of ORF2 RNA influenced productive infection, Neuro-2A cells were transfected with BoHV-1 genomic DNA. A BoHV-1 recombinant virus that contains the Lac Z gene inserted downstream of the gC promoter (BoHV-1 gCblue virus) was used for this study. The gCblue virus grows to similar titers, as wt BoHV-1 and β-Gal expression directly correlates with viral replication because the gC promoter is a late promoter, and its expression is low prior to viral DNA replication. gCblue DNA was used instead of infecting cells because VP16 and other regulatory proteins in the virion, bICP4 for example [38], diminish the stimulatory effects of DEX and the expression of regulatory genes on productive infection. Transfecting Neuro-2A cells with BoHV-1 DNA generally leads to a quiescent infection because the infection of Neuro-2A cells with infectious BoHV-1 yields approximately 10,000 fold less infectious virus relative to bovine cells [30]. As previously reported [23], transfection of Neuro-2A cells with gC-Blue, Krüppel-like transcription factor 15 (KLF15), and GR increased the number of β-gal+ cells approximately 10-fold relative to transfecting cells with just gC-Blue DNA (Figure 3A). The addition of DEX further stimulated the number of β-gal+ cells. This result was expected, because GR and KLF15 form a feed-forward transcription loop [39] to stimulate the IEtu1 promoter, which is a promoter that drives the expression of bICP0 and bICP4 [23]. Strikingly, the ORF2-Stop expression construct significantly reduced productive infection when 2 and 4 ug of the expression plasmid were included in cotransfection studies. In fact, the number of β-gal+ cells was less than the control, implying that ORF-2 RNA sequences also interfered with additional aspects of infection in Neuro-2A cells. We also examined the effects of increasing concentrations of ORF2-Stop on productive infection in Rabbit Skin (RS) cells because BoHV-1 grows efficiently in these cells. As expected, GR and KLF15 strongly stimulated productive infection, and DEX further enhanced productive infection (Figure 3B). Consistent with the results in Neuo-2A cells, ORF2-Stop significantly reduced productive infection. GR, KLF15, and DEX have no effect on gC promoter activity. Furthermore, ORF2 has no effect on gC basal promoter activity nor when GR, KLF15, and DEX are transfected with the gC promoter, suggesting that ORF2 and ORF2-Stop do not directly influence gC promoter activity (data not shown). 

GR-mediated transactivation is complicated, and there are many factors that regulate (positively or negatively) the efficiency of activating stress-induced promoters [40,41]. Additional studies tested whether ORF2 RNA influenced GR protein levels in transfected Neuro-2A cells. ORF2-Stop consistently reduced mouse GR from a co-transfected plasmid (Figure 3C). In summary, these studies suggested that ORF2 RNA sequences impaired productive infection and GR-mediated transactivation, in part, because it reduced steady-state GR protein levels from a co-transfected plasmid in transfected Neuro-2A cells. 

### 2.4. β-Catenin Reduces GR-Mediated Transactivation of the BoHV-1 IEtu1 Promoter

**Figure 4:**
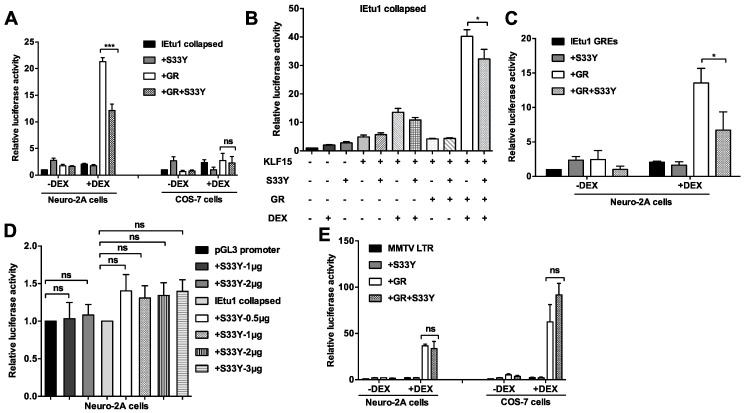
S33Y expression reduced GR-mediated transactivation of the IEtu1 promoter. **Panel** (**A**–**C**): Neuro-2A cells or COS-7 cells were cotransfected with the IEtu1 collapsed promoter construct containing the firefly luciferase reporter gene (0.5 μg) and where indicated plasmids that express GR (1.0 μg) or S33Y (1.0 μg). Where denoted, KLF15 (0.5 μg) and/or S33Y (1.0 μg). **Panel** (**D**): The empty pGL3-Promoter vector (0.5 μg) or IEtu1 promoter (0.5 μg) was cotransfected with a plasmid expressing the denoted concentration of S33Y. **Panel** (**E**): Neuro-2A or COS-7 cells were transfected with the MMTV-LTR promoter construct (0.5 μg), which is a plasmid that expresses GR (1.0 μg) and/or S33Y plasmid (1.0 μg). A plasmid that expresses Renilla luciferase (0.05 μg) was included in all transfections. To maintain the same amount of DNA in each sample, an empty vector was included in certain samples. Cells were incubated with 2% stripped FBS 24 h after transfection and, where indicated, cultures treated with DEX (10 μM). Dual-luciferase assay was performed as described in the Materials and Methods. The results are the average of three independent experiments, and error bars denote standard error. Student’s *T*-test was used for statistical analysis: not significant (ns); *, *p* < 0.05; ***, *p* < 0.001.

β-catenin is expressed in significantly more TG neurons during latency compared to uninfected TG neurons, and the ORF2 protein stimulates β-catenin+-dependent transcription [11,13,14]. GR activation correlates with BoHV-1 gene expression during reactivation and reduced β-catenin+ TG neurons. Hence, studies were performed to test whether β-catenin has the potential to impair stress-induced transcription. A Flag tagged β-catenin expression plasmid construct (S33Y) was used to test whether this gene reduced GR-mediated transactivation of the IEtu1 promoter. This construct has a β-catenin cDNA that encodes a protein with a serine to tyrosine mutation at amino acid 33 (S33Y), which interferes with β-catenin protein degradation [42]. Consequently, S33Y expression constitutively activates β-catenin dependent transcription. Neuro-2A and SV40 transformed monkey kidney (COS-7) cells were used for these studies because both cell lines are readily transfected and COS-7 cells do not express detectable levels of endogenous GR [43] (data not shown). Strikingly, GR-mediated transactivation of the IEtu1 collapsed promoter in Neuro-2A cells was significantly reduced by the S33Y construct when cultures were treated with DEX (GR+S33Y+DEX; Figure 4A, left panel). The maximum activity of the IEtu1 collapsed promoter (GR+DEX) in COS-7 cells was only 2.5-fold: thus, S33Y did not significantly influence promoter activity (Figure 4A, right panel), suggesting cell-type specific transcription factors were important for GR-mediated transactivation. We also tested whether the S33Y construct influenced GR+KLF15 cooperative transactivation of the IEtu1 collapsed promoter. The S33Y construct significantly reduced GR+KLF15+DEX mediated transactivation of the BoHV-1 IEtu1 collapsed promoter in Neuro-2A cells (Figure 4B). 

A 280-bp fragment that contains both GREs within the IEtu1 promoter cloned upstream of a minimal SV40 early promoter is designated IEtu1 GREs [22,23]. Previous studies demonstrated that GR and DEX consistently transactivated the IEtu1 GRES construct in Neuro-2A cells [22,23]. GR+DEX mediated transactivation of the IEtu1 GREs construct was also significantly reduced by the S33Y construct in Neuro-2A cells (Figure 4C). Conversely, the pGL3 promoter, which is the empty luciferase vector containing the SV40 minimal promoter, was not significantly affected by S33Y (Figure 4D). Furthermore, basal promoter activity of the IEtu1 promoter was not changed significantly when cotransfected with the S33Y plasmid. Interestingly, SS3Y expression did not significantly reduce MMTV LTR promoter activity in Neuro-2 and COS-7 cells using 1 ug S33Y (Figure 4E) and higher levels of S33Y (data not shown). These studies indicated that β-catenin (S33Y) reduced GR-mediated transactivation of the IEtu1 promoter but not the MMTV LTR in transfected Neuro-2A cells. This observation further suggested that cell-type specific factors were important for these studies and that all promoters are not impaired by the S33Y β-catenin expression construct. 

### 2.5. Wt β-Catenin Is Unstable in Neuro-2A Cells and Does Not Significantly Reduce IEtu1 Promoter Activity

**Figure 5:**
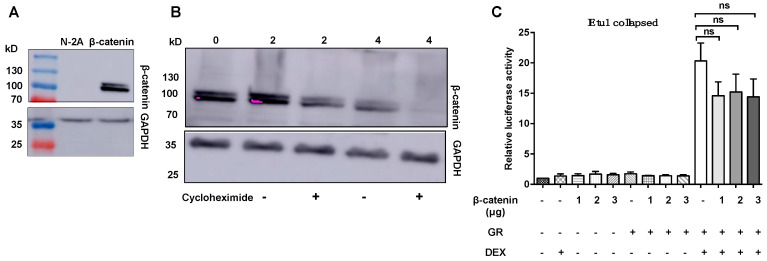
Wt β-catenin is unstable in Neuro-2A cells and does not interfere with GR-mediated transactivation of the IEtu1 promoter. **Panel** (**A**): Neuro-2A cells were transfected with a plasmid that expresses wt β-catenin (2.0 μg) or mock-transfected. Twenty-four hours after transfection, WCL was prepared and Western blots were performed using a β-catenin or GAPDH-specific antibody. Each lane contained 10 μg of protein WCL. **Panel** (**B**): Neuro-2A cells were transfected with wt β-catenin (2.0 μg). Twenty-four hours after transfection (denoted as 0), cultures were treated with cycloheximide denoted with a + symbol (100 μg/mL) for 2 h and 4 h to inhibit protein synthesis. Neuro-2A cells were lysed and processed for Western blot analysis to detect β-catenin or GAPDH. Molecular weight markers and the size of bands are denoted for **Panel** (**A**,**B**). **Panel** (**C**): Neuro-2A cells were transfected with IEtu1 collapsed promoter construct (0.5 μg), plasmid that expresses GR (1.0 μg), or wt β-catenin (1.0, 2.0, or 3.0 μg) and a plasmid expressing Renilla luciferase (0.05 μg). Empty vector plasmid was added to certain samples to maintain the same amount of DNA in each transfection. Neuro-2A cells were incubated with 2% stripped FBS, and 24 h following transfection, the designated cultures were treated with DEX (10 μM). At 48 h after transfection, cells were harvested, and protein lysate was subjected to a dual-luciferase assay. The results are the mean from three independent experiments. Statistical analysis was performed with the Student’s *T*-test: not significant (ns).

Endogenous β-catenin in Neuro-2A cells is not readily detected, except when a wt β-catenin expression construct is transfected into Neuro-2A cells [11,13] and (Figure 5A). Even when Neuro-2A cells were transfected with the wt β-catenin expression construct, the protein is nearly undetectable when cultures were incubated with cycloheximide for 4 h to block de novo protein synthesis, indicating that wt β-catenin was not stable in Neuro-2A cells (Figure 5B). While wt β-catenin reduced IEtu1 promoter activity, the effects were not significant (Figure 5C), presumably because wt β-catenin is unstable and thus was unable to significantly reduce IEtu1 promoter activity. 

## 3. Discussions

In this study, we provide evidence that RNA spanning ORF2 coding sequences impaired GR-dependent transcription of the IEtu1 promoter, MMTV LTR, and efficiency of productive infection. This finding was unexpected, because previous studies indicated that the multi-functional ORF2 protein plays an important role during the latency–reactivation cycle [8,27,44]. While these studies were all transient transfection studies and not performed in the context of a latent infection in cattle, we suggest that these findings are relevant to maintaining latency following low levels of stress. For example, chronic stress or high levels of acute stress frequently induces BoHV-1 reactivation from latency, which correlates with the ability of the synthetic corticosteroid DEX to induce reactivation, reduce LR-RNA levels [45], and reduce LR promoter activity [46]. While we suggest that these novel functions of the LR gene are important for maintaining a latent infection, additional cellular and viral components are likely to also play a significant role. For example, the canonical Wnt/β-catenin signaling pathway is likely to be important because it promotes neuronal survival, synaptic functions, and axon guidance [15,16,47]. Secondly, the anti-apoptosis functions of ORF2 expression promote the establishment and maintenance of latency by increasing the number of infected neurons that survive [36,48]. Thirdly, the ability of the two LR-encoded miRNAs to interfere with bICP0 protein expression would impair productive infection and promote the establishment and maintenance of latency [12]. Finally, we speculate that additional unknown cellular signaling pathways contribute to maintaining a latent infection in sensory neurons. 

Over-expression of the ORF2 protein in Neuro-2A cells increases Akt3 steady-state levels [11], but it has no effect on GAPDH, high mobility group AT-hook 1, and β-catenin protein levels [14]. Thus, it does not appear that the ability of ORF2 RNA to reduce steady-state levels of the GR protein was random. Interestingly, other non-coding RNAs interfere with GR-mediated transactivation. For example, the growth arrest-specific 5 (Gas5) non-coding RNA suppresses GR-mediated transactivation by acting as a decoy GRE: hence, this RNA competes for GR binding to DNA [49]. The 3′-untranslated region (3′UTR) of the GR transcript contains binding sites for several miRNAs [50]; interestingly, a subset of these miRNAs is abundantly expressed in the brain [51]. Since the GR construct used for this study contains the ORF but not the 3′UTR of the GR mRNA, we predict that ORF2 RNA sequences reduced GR steady-state protein levels by a novel mechanism. Considering there are multiple transcripts derived from the LR gene and many of these are alternatively spliced and not poly A+ [26,52], additional studies are necessary to fully understand how RNA sequences spanning ORF2 interfere with GR-mediated transactivation. 

These studies also demonstrated that a β-catenin mutant resistant to proteasome-dependent degradation (S33Y) interfered with GR-mediated transactivation of the IEtu1 promoter but not the MMTV LTR. Although the IEtu1 promoter and MMTV LTR contain functional GREs, the GR-dependent transactivation of specific promoters depends on novel coactivators and corepressors. For example, host cell factor 1 (HCF-1) is important for GR-mediated transactivation of the IEtu1 promoter but not the MMTV LTR in Neuro-2A cells [53]. Furthermore, KLF4 cooperates with GR to transactivate the bICP0 E promoter, but it interferes with GR-mediated activation of the MMTV LTR [54]. While it was clear that wt β-catenin did not significantly reduce IEtu1 promoter activity, we suggest that when the β-catenin destruction complex is not active, β-catenin directly or indirectly can impair certain promoters stimulated by stress. A recent study demonstrated that the serine/threonine protein kinase (Akt) impairs stress-induced transcription [55]. Since the Akt pathways and Wnt/β-catenin signaling pathways form a feed-forward signaling loop [56,57,58], we predict that the ability of β-catenin (S33Y) to impair stress-induced transcription is dependent on the Akt signaling pathway. 

In the context of the BoHV-1 latency–reactivation cycle, we propose that LR gene products do not directly stimulate reactivation from latency because the expression of LR gene products is significantly reduced during DEX-induced reactivation from latency [12,45,59]. We further suggest that LR gene products maintain a pool of latently infected neurons that can support reactivation from latency and produce infectious virus. These findings are supported by the finding that a LR mutant virus containing stop codons at the amino-terminus of ORF2 does not reactivate from latency following DEX treatment [27], in part because this mutant induced significantly more apoptosis in TG neurons during the establishment of latency [48]. The ability of ORF2 RNA sequences to impair the GR-mediated transactivation of viral promoters is predicted to actively promote the maintenance of latency in neurons by interfering with viral gene expression following stressful stimuli. Two micro-RNAs encoded by the LR gene also interfere with bICP0 protein expression and are abundantly expressed during latency but not reactivation [12]. These two micro-RNAs do not overlap with ORF2 coding sequences. The majority of LR-RNA is not poly-adenylated and may not be translated into a functional protein [8], which implies that additional non-coding RNAs are expressed during latency. The expression of multiple LR gene products that are abundantly expressed during latency has made it difficult to dissect which LR gene functions are crucial for regulating the latency–reactivation cycle. Constructing additional LR gene-specific mutants is further complicated because LR coding sequences overlap most of the bICP0 coding sequences. 

## 4. Materials and Methods 

### 4.1. Cells, Plasmids, and Viruses

Murine neuroblastoma cells (Neuro-2A; CCL-131) and COS-7 cells were obtained from American Type Culture Collection ATCC (Manassas, VA USA) and grown in Minimal Essential Media (MEM; Life Technology, Carlsbad, CA, USA) supplemented with 10% fetal bovine serum (FBS), penicillin (10 U/mL), and streptomycin (100 μg/mL). Rabbit Skin (RS) cells were obtained from Steve Wechsler (UC-Irvine, CA, USA) and cultured as above.

The mouse GR construct used for these studies was obtained from Dr. Cidlowski (NIEHS, Research Triangle Park, NC, USA). The ORF2 expression construct and ORF2-Stop was generated in pCMV-Tag-2B vectors (Stratagene; La Jolla, CA, USA) as described previously [11,36]. These plasmids only contain ORF2 coding sequences from BoHV-1 genomic sequences and no untranslated regions. A Flag epitope is present at the N-terminus of ORF2, and the human IE CMV promoter drives its expression. For ORF2-Stop, the genomic sequences of ORF2 were synthesized. The first in-frame ORF2 AUG was deleted and replaced with three stop codons such that translation will not occur in all three reading frames; this fragment was synthesized by GenScript (Piscataway, NJ, USA) and then cloned into the pCMV-Tag-2B vector. The IEtu1 collapsed promoter was synthesized by GenScript, and the promoter sequences are inserted at KpnI and HindIII restriction sites of the pGL3-Basic Vector (Promega, Madison, WI, USA) as previously described [60]. This construct contains the two GREs but is missing nucleotides 109,092–109,861 at the 5′-terminus. The 3′-terminus of the IEtu1 collapsed construct is 108,807, and the 5′-terminus is 109,861. A 280 bp fragment that contains the two GREs in the IEtu1 promoter was previously described [23]. These sequences were synthesized by GeneScript and cloned into the pGL3-Promoter Vector (Promega) at unique KpnI and XhoI restriction enzyme sites (IEtu1 GREs). The MMTV LTR luc construct (plasmid #91689; Addgene; Watertown, MA, USA) was obtained from Dr. J. Rosen. 

A BoHV-1 mutant containing the β-Gal gene in place of the viral gC gene was obtained from Dr. S. Chowdury (LSU School of Veterinary Medicine) (gCblue virus) and stocks of this virus are grown in MDBK cells. The gCblue virus grows to similar titers as the wt parental virus and expresses the Lac Z gene. Procedures for preparing genomic DNA were described previously [44].

### 4.2. Dual-Luciferase Assay

Neuro-2A or COS-7 cells were seeded into dishes containing MEM with 10% fetal bovine serum (FBS) at 24 h prior to transfection. At 2 h before transfection, cells were cultured with antibiotic-free medium containing 2% stripped FBS and DEX added as indicated in the Figure Legends. All plasmids were transfected using Lipofectamine 3000 transfection reagent (L3000075; Invitrogen, Carlsbad, CA, USA) according to the manufacturers’ instructions. Cells were cotransfected with the designated plasmids and a plasmid carrying Renilla luciferase under the control of a minimal herpesvirus thymidine kinase (TK) promoter (50 ng). To maintain equal plasmid amounts in the transfection mixtures, an empty expression vector was added as needed. At 48 h after transfection, cells were harvested, and protein lysate subjected to a dual-luciferase assay by using a commercially available kit (catalog number E1910; Promega; Madison, WI, USA) according to the manufacturer’s instructions. Luminescence was measured using a GloMax 20/20 luminometer (catalog number E5331; Promega).

### 4.3. Western Blot Studies 

For Western blot studies, cells were collected, washed once with PBS, and then lysed in RIPA buffer (50 mM Tris-HCl, pH 8, 150 mM NaCl, 1% Triton X-100, 0.5% sodium deoxycholate, 0.1% SDS) with protease and phosphatase inhibitors (Thermo-Scientific, Waltham, MA, USA). Samples were boiled in Laemmli sample buffer for 5 min, and all samples were separated on an 8% or 10% SDS–polyacrylamide gel. Immunodetection of the respective proteins was performed using the antibodies described above. The anti-Flag monoclonal antibody (Sigma, F1804), β-catenin antibody (LifeSpan Biosciences; LS-C21245, Seattle, WA, USA), and GR antibody (Cell Signaling 3660S, Danvers, MA, USA) were used for these studies. 

## Data Availability

The data that support the findings of this study are available from the corresponding author upon reasonable request.
